# Cell-Based Veterinary Pharmaceuticals – Basic Legal Parameters Set by the Veterinary Pharmaceutical Law and the Genetic Engineering Law of the European Union

**DOI:** 10.3389/fvets.2016.00101

**Published:** 2016-11-30

**Authors:** Timo Faltus, Walter Brehm

**Affiliations:** ^1^Faculty of Law, Economics and Business, Martin-Luther-University Halle-Wittenberg, Halle, Germany; ^2^Faculty of Veterinary Medicine, Leipzig University, Leipzig, Germany

**Keywords:** veterinary pharmaceuticals, cell therapy, stem cells, blood products, ACS, IRAP, ACP, PRP

## Abstract

Cell-based therapies have been in use in veterinary medicine for years. However, the legal requirement of manufacturing, placing on the market and use of cell-based veterinary pharmaceuticals are not as well developed as the respective requirements of chemical pharmaceuticals. Cell-based veterinary pharmaceuticals are medicinal products in the sense of the pharmaceutical law of the European Union (EU). For that reason, such medicinal products principally require official approval for their manufacture and an official marketing authorization for their placement on the market before being used by the veterinarian. The manufacture, placing on the market, and use of cell-based veterinary pharmaceuticals without manufacturing approval and marketing authorization is permitted only in certain exceptional cases determined by EU and individual Member State law. Violations of this requirement may have consequences for the respective veterinarian under criminal law and under the code of professional conduct in the respective Member State. The regular use of cell-based veterinary pharmaceuticals within the scope of a therapeutic emergency as well as the import of such veterinary pharmaceuticals from non-European countries for use in the EU are currently out of the question in the EU because of a lack of legal bases. Here, we review the general legal requirement of manufacturing, placing on the market, and use of cell-based veterinary pharmaceuticals within the EU and point out different implementations of EU law within the different Member States.

## Introduction

Cell-based therapies have been available in veterinary medicine for years and are currently experiencing a growing demand. Hereby, they do not only play an import role within the preclinical testing of cell-based pharmaceuticals for human use but rather as cell-based therapies for animals *per se* (1, 2). However, such cell-based therapies can, especially when performable in mammals, serve as an example for the development of respective cell-based therapies for human use. This is due to the fact that in general “first in animal” studies can be conducted easier than “first in man” studies in regard to legal and actual requirements. Veterinarians across the European Union (EU) already offer such cell-based therapies to their clients and prepare the necessary cell-based therapy products either themselves or obtain them from suppliers in their respective member state or from abroad. Despite their actual dissemination and meaning, also for the development of pharmaceuticals for human use, (stem) cell-based pharmaceuticals for veterinary use have been widely ignored by legal practice, and no special-law provisions have been issued compared to (stem) cell-based pharmaceuticals for human use. The existing EU legislation on veterinary medicines is, in any case, incomplete and leaves too many loopholes for unproven cell-based pharmaceuticals for veterinary use, which then can also serve as false examples for the development of cell-based pharmaceuticals for human use. Therefore, the current EU and national legislations on veterinary medicines need to be reformed so as to bring about such legislative and actual improvements, which also reflect the meaning of cell-based pharmaceuticals for veterinary use for the development of cell-based pharmaceuticals for human use.

## Treatment Models

Since the first publication of a particular case in 2003, for example (3), tendon diseases of horses have been treated with mesenchymal stem cells of various origins, and the cell product or the manufacture of the cell product has been offered by a range of companies. The first enterprise of this type was VetCell^®^ in Great Britain, which offered the treatment of bone marrow samples. The attending veterinarian extracts bone marrow, sends it to the company, which processes it in its laboratory and returns a therapy product to the veterinarian that the latter then administers. Veterinarians across the EU have been offering their clients such therapies as well, by either preparing the necessary cell-based therapy products themselves or obtaining them from domestic or foreign third parties. Various situations in the preparation and use of veterinary pharmaceuticals based on cells are currently being observed:

– Autologous therapies by the veterinarian involving veterinary pharmaceuticals prepared by the veterinarian: the attending veterinarian extracts samples from the patient (e.g., blood, bone marrow, or fat) and processes this material in his medical office for the purpose of administering the resulting veterinary medicinal product (e.g., cell product) to the same patient.
– Autologous therapies by the veterinarian involving veterinary pharmaceuticals not prepared by the veterinarian: the attending veterinarian extracts samples from the patient (see above) and sends them to a laboratory, which then processes the material and sends to the attending veterinarian a veterinary medicinal product (e.g., cells in suspension) for administration to the same patient.
– Allogenic therapies by the veterinarian involving veterinary pharmaceuticals prepared by the veterinarian: the attending veterinarian extracts samples from a donor animal (e.g., blood, bone marrow, and fat) and processes this material in his medical office for the purpose of administering the resulting veterinary medicinal product (e.g., cell product) to a recipient animal (patient).
– Allogenic therapies with finished veterinary medicinal products not prepared by the veterinarian: the attending veterinarian obtains a veterinary medicinal product (e.g., cell therapy product) from a provider and administers it to his/her patient.

Although in all treatment settings mentioned above the cell-based component is at the center of the therapy, there are fundamental differences in the legal handling of the individual settings. The manufacture, import, and use of cell-based veterinary pharmaceuticals have to comply with EU and respective national pharmaceutical law just like other medicinal products. Violations of pharmaceutical law may be prosecuted and may have consequences under the code of professional conduct for the veterinarian (suspension or even loss of medical license) where this is provided for in the individual Member State legislation. Compared to the human domain, the veterinary pharmaceutical law is marked by a more complex principle of exceptions to regulations. In addition, there are specific provisions for individual animal species, regulations that do not exist in this form with respect to humans. This makes the practical manageability of the veterinary pharmaceutical law more difficult for the legal expert and particularly for the veterinarian in practice but does not release them from complying with the respective regulations.

## Categorization of (Stem) Cell-Based Animal Therapies Under Pharmaceutical law

Cell-based veterinary therapeutics are medicinal products in the sense of the pharmaceutical law of the EU (Art. 1 No. 2 Directive 2001/82/EC) and hereby defined as any substance or combination of substances presented as having properties for treating or preventing disease in animals; or any substance or combination of substances, which may be used in or administered to animals with a view either to restoring, correcting, or modifying physiological functions by exerting a pharmacological, immunological, or metabolic action, or to making a medical diagnosis. Whether the physiological effect of cell-based forms of therapy results from a pharmacological, immunological, or metabolic effect is of no consequence since such therapy products, at least in the sense of Art. 1 No. 2 Directive 2001/82/EC, are substances or preparations from substances designated for use in or on the animal body and intended as remedies with properties for curing, relieving, or preventing diseases or disease symptoms. This eliminates the need to resolve the extent to which the cell–cell interactions made use of in a (stem) cell-based animal therapy can be attributed to a pharmacological, immunological, or metabolic effect. Respective definitions can be found within the Medicinal Products Acts of the EU Member States, for example, within Sec. 2 German Medicinal Products Act.

(Stem) Cell-based veterinary pharmaceuticals are not, at any rate, medical devices in the sense of the directives of EU law regarding the definition and regulation of medical devices since EU law regarding medical devices only applies to medical devices for human use. In other words, there is no specific EU legislation covering medical devices for veterinary use. This loophole in veterinary legislation can therefore lead to doubtful and/or unproven therapy offers and should be addressed by future amendments of veterinary legislation. Furthermore, medicinal devices are characterized by the fact that these are items *used* within the scope of a therapeutic application. Substances that medicinal products are composed of, by contrast, are *spent* or *processed* (4). However, the therapeutically administered (stem) cells do not permanently remain in or on the animal body because, like principally all cells, they have a limited life span that is shorter than the life span of the organism as a whole to which they are administered. The administered cells therefore develop their therapeutic effect during the time between their administration and their dissolution brought about, probably among others, by cell–cell interactions or by substances released by the administered cells. By contrast, a physico-mechanically acting therapeutic object principally remains in the body of the patient until it is removed (absorbable suture material nonetheless is not a medicinal product because its principal intended effect is physical rather than specific to a medicinal product, cf. Art. 1 para. 2 lit. a) Directive 93/42/EEC). Therefore, therapeutically used cells constitute consumable substances or substances that are processed (see Requirement of Official Authorization for (Stem) Cell Isolation), which means that cells can principally have the characteristic of a medicinal product.

Furthermore, cell-based forms of therapy in the veterinary sector are not tissue in the sense of the EU Tissue Directive 2004/23/EC since that directive lays down standards of quality and safety only for human tissues and cells intended for human applications in order to ensure a high level of protection of human health (Art. 1). These special provisions of tissue law must thus not be applied to animal tissue used on or in an animal. Additionally, until today, there is no specific EU legislation governing cell-based medicinal products for veterinary use. However, the EU legislation regarding medicinal products for veterinary use is currently subjected to a reform process, which is supposed to cover cell-based medicinal products for veterinary use with specific provisions in the future (cf. Conclusion and Outlook).

## Possible Exception from the Application of Pharmaceutical Law

The fact that a product is a medicinal product in the sense of the EU pharmaceutical law is not to be confused with the issue of whether pharmaceutical law applies. The Community code for veterinary pharmaceuticals, Directive 2001/82/EC, first defines in Art. 1 No. 2, the substances and substance preparations, which are considered veterinary pharmaceuticals. Then, in Art. 2 No. 1, the Community code stipulates that this Directive shall apply (only) to veterinary medicinal products, including pre-mixes for medicated feeding stuffs, intended to be placed on the market in Member States and prepared industrially or by a method involving an industrial process. Since the terms “*prepared industrially*” and “*industrial process*” have not been defined by EU law or jurisdiction yet, there is no EU-harmonized definition for such processes. Therefore, regarding (stem) cell-based veterinary medicinal products prepared in-house, i.e., within the practice of the veterinarian, it is unclear what would be the minimum production quantity to have an “industrial preparation” and/or an “industrial process” in legal terms to cover such in-house preparations by the scope of the Community code for veterinary pharmaceuticals. However, in the absence of the placing on the market in the sense of the Community code for veterinary pharmaceuticals, which means that only the manufacturer (e.g., the veterinarian) and its directly supervised and bound by instructions staff, but no third party such as another independent veterinarian or the owner of the respective animal has the power of disposal for the (cell-based) pharmaceutical, such not placed on the market veterinary pharmaceuticals are thus not be governed by the regulations of the Community code for veterinary pharmaceuticals in regard to an official marketing authorization. As a consequence, there is no requirement by the Community code in the Member States of the EU to have a marketing authorization for such (cell-based) pharmaceuticals produced in-house within the veterinarian’s practice. Therefore, the use of in-house prepared pharmaceuticals is typically permissible within the EU Member States if nothing else is stated within individual Member State law. For example, in Germany, such in-house prepared pharmaceuticals for veterinary use do not need a marketing authorization according to the German Drug Law. In any case, this exemption from the applicability of the Community code for veterinary pharmaceuticals is not equivalent with a statement concerning the therapeutic effect of the respective pharmaceutical. However, on the one hand, the therapeutic effect of pharmaceuticals that are placed on the market is regularly proven by preclinical and clinical trials, on the other hand, clinical trials therefore are typically connected to the official marketing authorization. Since in-house prepared (cell-based) pharmaceuticals for veterinary use do not need an official marketing authorization, they are also not bound to the legal requirements of being tested within clinical trials to examine and prove their therapeutic effect under scientific, statistical standards. Therefore, until today, claims regarding the therapeutic effect of in-house (cell-based) pharmaceuticals should be questioned and checked whether they are based on proper clinical trials. In any case, the veterinarian is obliged to inform the animal owner about this pharmaceutical character of in-house produced (cell-based) pharmaceutical to enable the animal owner in the context of an informed consent to decide whether he/she wants this kind of treatment for the animal and to avoid possible liability risks.

Additionally, one must not confuse the provisions to receive a marketing authorization with the additional need to receive a manufacturing license for the respective medicinal product. Regularly, both the marketing authorization and the manufacturing license are mandatory to manufacture and market the respective medicinal product. But, within the in-house preparation settings, there can be additional exceptions regarding the need for a manufacturing license. Since the Community code for veterinary pharmaceuticals only applies to veterinary medicinal products, including pre-mixes for medicated feeding stuffs, intended to be placed on the market in Member States and prepared industrially or by a method involving an industrial process (cf. above); initially, there is also no requirement by the Community code in the EU Member States to connect the in-house preparation of such (cell-based) veterinary medicinal products to a manufacturing license. However, this only concerns (cell-based) veterinary pharmaceuticals prepared and administered by the veterinarian himself/herself (cf. Art. 3 para. 2 lit. a) Directive 2001/82/EC) since these medicinal products are not dispensed to third parties. However, these legal requirements of the Community code for veterinary pharmaceuticals lead to different legislations regarding in-house (cell-based) veterinary pharmaceuticals within the Member States of the EU. For example, such in-house (cell-based) veterinary pharmaceuticals can be prepared and administered without a manufacturing and/or marketing authorization license in Germany, whereas in other Member States, veterinarians need a manufacturing license, which then can also be connected to the compliance with the Good Manufacturing Practice (GMP) that is among others a matter of additional personnel and costs. Therefore, veterinarians are called upon to check their individual Member State law regarding the requirements for in-house preparing (cell-based) veterinary pharmaceuticals to avoid criminal prosecution and/or negative consequences on basis of the code of professional conduct where this is provided for in the individual Member State legislation.

## Requirement of Official Authorization for (Stem) Cell Isolation

In any case, placing on the market is planned by the manufacturer or takes place actually, i.e., the change in power of disposal for the respective (cell-based) pharmaceutical, this has also an effect on the initial (stem) cell isolation from the animal. In these cases, concerning the manufacture of active substances and/or medicinal products in the sense of pharmaceutical law, the biopsy of cells and tissue from animals is covered, among others, by Art. 44, 50 lit. f), 50a Directive 2001/82/EC and thus principally requires authorization in the Member States. One reason why the isolation of animal cells for therapeutic use in the veterinary sector requires official authorization according to Art. 44, 50a Directive 2001/82/EC, is because the animal cells to be biopsied are active substances and not just source or raw material, which can principally be obtained without authorization. To be considered as an active substance requires that the material in question is a substance in the sense of the pharmaceutical law because the term “active substance” goes back to the term “substance” used in pharmaceutical law and supplements it. Animal (stem) cells fit the term “substance” used in pharmaceutical law because, pursuant to Art. 1 No. 4, 2nd bullet point, Directive 2001/82/EC, they are body components of animals, including cells (5). It follows that these substances, if additional requirements are fulfilled, are principally suited to be or be made into an active substance.

The term “*active substance*” is not defined for veterinary medicinal products within the Community code. But the term can be interpreted under legal aspects in compliance with Art. 1 No. 3a of the Community code for medicinal products for human use (Directive 2001/83/EC). Since both directives refer to “*active substance*” and both directives furthermore concern the same legal matter, namely, the regulation of the pharmaceutical sector, such a comprehensive interpretation is possible. Based on the substance concept in pharmaceutical law and with due regard to the term “active substance,” an active substance in the sense of the pharmaceutical law thus is a substance intended to be used as an active pharmaceutical ingredient in the manufacture of (veterinary) medicinal products or to become an active pharmaceutical ingredient of the (veterinary) medicinal products when used in the manufacture of (veterinary) medicinal products. These prerequisites for the term “active substance” do not conflict with the possibility that, with respect to animal (as well as human) cells, the biopsied animal cells may have to be modified and/or cultivated *in vitro* by cell and tissue cultivation processes before being used therapeutically on animals.

If the biopsied animal (stem) cells were used in therapy without further processing, these cells would also be an active substance in the sense of pharmaceutical law because these cells would already be an active pharmaceutical ingredient of a medicinal product. Nothing else can then apply to cells processed after the biopsy, since the cells to be biopsied are intended, when used in the manufacture of pharmaceuticals, to become an active pharmaceutical ingredient of the respective medicinal product. This is also consistent with the fact that animal organs are not considered active substances, if substances are first to be extracted from these organs or from the individual cells of these organs for later use in the production of medicinal products.^1^ The key difference between acknowledging the property of active substance with respect to animal cells to be biopsied and denying the property of active substance to animal organs lies in the fact that the cells that are to be used therapeutically are used as such and become part of the finished therapy product, whereas in the case of organs, it is only one component that is extracted, while the organ as a whole, or the cells in this case, is/are not present in the therapeutic product. If the organ were rated to be an active substance, then the biopsy of cells for therapeutic use would by implication classify the living organism as a whole as an active substance.

Manufacturing (of the active substance) means, *inter alia*, the acquisition of an active substance; in the case of cells “acquisition” must mean cell removal, i.e., biopsy. Pursuant to Art. 50 lit. f) Directive 2001/82/EC the manufacture and thus cell acquisition shall be performed under the conditions of GMP. In the assessment of Directive 2001/82/EC, the veterinarian, however, does not need an authorization for cell removal, if he/she isolates the cells under his/her immediate technical responsibility for the purpose of personal application to animals he/she is treating, since there is no placing on the market involved. It is therefore irrelevant whether the treatment regimens are autologous or allogenic as long as the same veterinarian is exclusively in power of disposition related to the biopsied cells and the in-house prepared cell-based pharmaceuticals respective as well as related to the administration of this pharmaceutical and as long as this remains a non-industrial activity. However, this requirement of the Directive has been implemented differently within the Member States, i.e., in Germany such in-house preparations do not need manufacturing licenses, whereas other Member States require a license even for in-house prepared (cell-based) pharmaceuticals, which are not placed on the market (cf. Possible Exception from the Application of Pharmaceutical Law). In any case, failure to comply with the authorization requirement can routinely be prosecuted as a criminal and/or administrative offense in the respective Member States if a manufacturing license is needed in the respective Member State or if individual Member State law does not allow in-house medicinal preparation at all.

## Processing of Animal (Stem) Cells Requiring Authorization

Pursuant to Art. 44 para. 1 Directive 2001/82/EC, the manufacture of a (stem) cell-based therapeutic product for the veterinary sector constitutes the manufacturing of a (veterinary) pharmaceutical, which principally requires authorization. Art. 44, 50 lit. f) Directive 2001/82/EC states that the manufacture of veterinary medicinal products must comply with the conditions of GMP. Here, too, the special provisions of the Tissue Directive 2004/23/EC of the EU are not applicable in the veterinary sector because the Tissue Directive, as stated by its Art. 1, is pertinent only to the therapeutic use of human tissues on humans. Responsible for issuing the authorization are the authorities within the respective Member State, in Germany, for example, the authorities of the federal state in which the manufacture actually is to take place.

With respect to the manufacture of a cell-based veterinary pharmaceutical, the veterinarian also does not need an official authorization in the sense of the Art. 44, 50 lit. f) Directive 2001/82/EC for the preparation in autologous and allogenic treatment regimens, if he/she processes the previously removed cells under his/her immediate technical responsibility for the purposes of the treatment he/she is performing (cf. Art. 3 para. 2 lit. a Directive 2001/82/EC) as long as the same veterinarian is exclusively in power of disposition related to the biopsied cells and the in-house prepared cell-based pharmaceuticals respective as well as related to the administration of this pharmaceutical and as long as this remains a non-industrial activity. If, in a given case, the veterinarian removes (stem) cells from an animal and processes these cells and then administers these cells to an animal, an official authorization is not needed, as stated in Art. 44, 50 lit. f) Directive 2001/82/EC. However, this requirement of the EU Directive 2001/82/EC has been implemented differently within the Member States, so that in some states such as Germany, no license is necessary for the treating veterinarian to prepare such cell-based pharmaceuticals in-house, whereas in other member states (cf., for example, legal situation in Belgium), the treating veterinarian is not allowed to prepare his own medicinal product. In any case, conversely it also means that the veterinarian does need such an authorization, if he/she removes and processes the cells and then releases the cells from his/her veterinary area of delegation. Failure to comply with the authorization requirement regarding the manufacture of medicinal products can be routinely prosecuted in the Member States where this is provided for in the individual Member State legislation. For example, in Germany, violations can be punished under Section 96 No. 4 German Medicinal Products Act by imprisonment of up to 1 year or by a fine.

## Marketing Authorization Requirement for (Stem) Cell-Based Veterinary Pharmaceuticals

As veterinary pharmaceuticals in the sense of Art. 1 No. 2 Directive 2001/82/EC, veterinary pharmaceuticals in the EU principally require marketing authorization as per Art. 5 para. 1 Directive 2001/82/EC. In the absence of marketing authorization, the veterinary pharmaceutical may not be placed on the market. Since the veterinary pharmaceutical law is harmonized within the EU law by Directive 2001/82/EC (Community code for veterinary pharmaceuticals), this requirement is applicable in all EU Member States. There are various legally named exemptions from the marketing authorization requirement. Such legally named exemptions are not to be confused with the abovementioned (cf. Possible Exception from the Application of Pharmaceutical Law) questions of the general applicability of the Community code for veterinary pharmaceuticals due to missing industrial preparation/industrial processes and/or the missing placing on the market in legal terms. If, however, such a legally named exemption exists, the respective veterinary pharmaceutical can be placed on the market and used without official marketing authorization. In the assessment of Art. 3 para. 2 Directive 2001/82/EC that applies, among others, to veterinary pharmaceuticals produced in pharmacies for individual animals or animals of a particular holding. However, in accord with the assessments of the regulations regarding a therapeutic emergency (see below), this exemption may only be claimed if, among others, an authorized medicinal product is not available for the respective animal species or the respective field of application and if the required medical care of the animals would otherwise be seriously jeopardized. The efficacy of cell-based pharmaceuticals in the veterinary sector still needs to be demonstrated and currently available authorized medicinal products ensure medical care of most patients to a great extent. Therefore, regarding cell-based pharmaceuticals, therapeutic emergency should currently be questioned and only applied when thoroughly investigated and justified. This situation may change, however, depending on clinically proven indications for the therapeutic use of cell-based products. In any case, a veterinarian may use an alternative yet unproven cell-based therapy only in case (conservative) registered therapies are verifiably ineffective.

An additional exemption from the marketing authorization requirement can arise within the scope of cell-based on-site treatment regimens. According to the assessment of Art. 2 para.1; 3 para. 2 Directive 2001/82/EC, the marketing authorization requirement concerns only medicinal products placed on the market. Without placement on the market, there can be no requirement of marketing authorization. But a placement on the market does not exist when the veterinarian uses the veterinary pharmaceutical in question on an animal. What is missing for the placement on the market is the concession of the power of control over the pharmaceutical concerned to third parties apart from the veterinarian (8–10).^2^ With respect to cell-based veterinary pharmaceuticals, the veterinarian therefore – in autologous as well as in allogenic treatment models – has to remove the cells him/herself (within the scope of his/her veterinary freedom of delegation), process them him/herself, if applicable, and then use them him/herself on the animal in order not to require authorization under pharmaceutical law and marketing authorization for these activities. This does therefore also mean that cell-based veterinary pharmaceuticals not prepared by the veterinarian him/herself, i.e., which the veterinarian acquires from third parties, principally require marketing authorization regardless of whether they were obtained in Germany or abroad. Therefore, the outsourcing of the preparation of the (stem) cell-based pharmaceutical after the biopsy from the veterinarian’s practices to a third-party manufacturer can only be in compliance with the requirements of EU pharmaceutical law, if the third-party manufacturer holds an official manufacturing license according to EU law. Additionally, the veterinarian needs a license for the biopsy itself, if she/he supplies a third party with cells to manufacture a cell-based medicinal product (cf. Requirement of Official Authorization for (Stem) Cell Isolation). The license for the veterinarian is mandatory, to ensure compliance with the GMP standard. This supplying action of the veterinarian is therefore different from the situation, when the veterinarian just takes a biopsy to analyze the cells within a diagnostic procedure. The veterinarian does not need an extra license for such diagnostic related biopsies.

## Competence for Marketing Authorization

Which authority is competent for authorizing the marketing of a veterinary pharmaceutical depends on the type of actual veterinary pharmaceutical. The European Commission, for example, advised beforehand by the European Medicines Agency (EMA), is competent under the terms of Art. 3 para. 1 Regulation (EC) No. 726/2004 for issuing the marketing authorization for veterinary medicinal products manufactured by certain biotechnological methods. Veterinary medicinal products manufactured, among others, by recombinant DNA technology or processes of the controlled expression of genes coding for biologically active proteins in prokaryotes and eukaryotes, including transformed mammalian cells, therefore have to receive mandatory centralized marketing authorization from the Commission. If neither of these techniques is applied and if the treatment does not serve to improve agro-technical performance, for example, the marketing authorization procedure of the respective Member State is applicable. Relevant in Germany is the procedure as per Section 21 German Medicinal Products Act. The competent agency for the marketing authorization of such cell-based veterinary pharmaceuticals thus is – under the terms of Section 77 para. 3 German Medicinal Products Act – the Federal Office for Consumer Protection and Food Safety (BVL).

## Prescription Requirement for Cell-Based Veterinary Therapeutic Products

Cell-based veterinary pharmaceuticals are subjected to medical prescription. One reason why the classification of cell-based veterinary pharmaceuticals as prescription (veterinary) pharmaceuticals is of significance under legal aspects is the fact that violations with respect to the use or marketing of prescription pharmaceuticals entail stiffer penalties than those involving non-prescription pharmaceuticals.

Within the EU, the prescription requirement for cell-based veterinary pharmaceuticals follows from Art. 67 lit. c) Directive 2011/82/EC. It makes veterinary pharmaceuticals available by veterinary prescription only if, among others, the product is intended for the treatment of diseases that require prior exact diagnosis or if its use may have consequences that would hamper or interfere with the subsequent diagnostic or therapeutic measures. The stem cell therapy of a horse’s tendon disease, for example, always involves veterinary diagnostics (clinical diagnostics, ultrasound diagnostics) that are necessary in any case to determine the localization, the extent and the age of the damage in order to be able to establish a specific therapy plan and to administer the cell therapy product pointedly and effectively.

## Cell-Based Pharmaceuticals Not to be Used in Non-Food-Producing Animals within the Scope of a Therapeutic Emergency

The provisions regarding the use of pharmaceuticals within the scope of a therapeutic emergency have been harmonized within the EU by Directive 2001/82/EC and currently leave no room for the use of cell-based pharmaceuticals in the veterinary sector. In a given case, the veterinarian may administer, dispense, or prescribe medicinal products under Art. 10 Directive 2001/82/EC that have not been authorized for the respective therapeutic indication and/or respective animal species, if the medical care of the animal to be treated would otherwise be jeopardized and a direct or indirect threat to the health of humans and animals is not to be feared. But the justification for these exemptions stated here remains to be demonstrated (cf. Marketing Authorization Requirement for (Stem) Cell-Based Veterinary Pharmaceuticals).

Art. 10 lit. a) Directive 2001/82/EC permits the treatment of the animal in question only with a (cell-based) veterinary pharmaceutical that is authorized in the respective Member State for another therapeutic indication or for another animal species. However, such cell-based veterinary pharmaceuticals do (currently) not exist in Germany. Even if they existed, it is doubtful whether the necessary medical care of the animals would be seriously jeopardized without their use (see above). Without such a hazard, however, the use in the sense of Art. 10 Directive 2001/82/EC is not admissible. In the example of the sinew therapy, a purely conservative therapy could be used instead of the cell therapy. While it might not have the same optimized therapeutic effect, this approach would hardly pose a serious risk to the patient. In general, only in case (conservative) registered therapies are ineffective, a veterinarian may use an alternative unproven (cell-based) therapy, whereas he/she is obliged to inform the owner of the animal about the scientific status of the respective unproven pharmaceutical to avoid the risk of liability.

Based on the exception stipulated in Art. 10 lit. b) Directive 2001/82/EC, medicinal products authorized for human use and (other) medicinal products authorized in a Member State of the EU or another signatory of the Agreement on the European Economic Area for use on animals can also be prescribed, administered, or dispensed to animals within the scope of a therapeutic emergency in the veterinary sector. In the area of cell-based types of therapy, for example, it would be conceivable that a respective cell-based pharmaceutical authorized for the treatment of cartilage defects of humans (such marketing authorizations exist) is used to treat the joint disorder of a dog. This is another case where the optimized treatment of the dog could possibly be compromised if it did not include the use of cell-based pharmaceutical for human use, but the animal would most likely not be seriously harmed.

Finally, according to Art. 10 lit. c) Directive 2001/82/EC, the veterinarian may, as an exception, treat the animal concerned with a veterinary medicinal product prepared extemporaneously by a person authorized to do so under national legislation in accordance with the terms of a veterinary prescription.

The manufacture of such cell-based prescription pharmaceuticals for animals will fail not least for practical reasons. While pharmacies are commonly authorized to prepare (veterinary) medicinal products, they are just as commonly technically unequipped to prepare such cell-based veterinary medicinal products. However, the exception also requires that the medical care of the animals be in serious jeopardy without such a prescription pharmaceutical. Depending on national law, the veterinarian may possibly be authorized to prepare such cell-based veterinary pharmaceuticals. In Germany, for example, this is permitted only to a limited extent. The preparation of a cell-based veterinary pharmaceutical by a veterinarian would only be an option, according to Section 56a para. 2 No. 4; Section 13 para. 2 Sentence 1 No. 3 lit. d) German Medicinal Products Act, if an authorized cell-based finished medicinal product existed from which the veterinarian in his/her veterinary dispensary would prepare a (new) medicinal product, possibly with the use of non-active pharmaceutical ingredients. The veterinarian thus first has to examine again whether, without the manufacture and use of a medicinal product manufactured and administered this way by the veterinarian the medical care of the concrete animal would be seriously compromised. Only then can a manufacture and use even be considered. If that is the case, the veterinarian then has to check whether such authorized cell-based finished medicinal products exist. For the veterinary sector, this is currently not the case, but individual cell-based medicinal products for human use have already been authorized.

It is important to note that in all Member States of the EU the veterinarian is not free to select a particular regulation from among the abovementioned exceptions of Art. 10 Directive 2001/82/EC that can be found in the legal systems of the respective Member States; instead, he/she has to follow the therapy cascade of Art. 10 stipulated by the law itself, which means examining first whether the exception as per Art. 10 lit. a) is pertinent. Only if this is not the case, he/she may resort to the regulation of Art. 10 lit. b). If this is not met either, he/she may use medicinal products as per Art. 10 lit. c).

Violations of the requirements of pharmaceutical law with respect to the handling of prescription veterinary pharmaceuticals in a therapeutic emergency are commonly prosecuted under national criminal law where this is provided for in the individual Member State legislation as it is in Germany.

## No Specific Regulations for Non-Food-Producing Equidae

The use of cell-based veterinary pharmaceuticals in Equidae follows the provisions pertaining to classical medicinal products for use in non-food-producing Equidae. This means that such Equidae may be treated with any medicinal product, including a cell-based veterinary pharmaceutical, as long as this is in compliance with the remaining provisions of pharmaceutical law (11).

## Import of Cell-Based Veterinary Pharmaceuticals into the European Union

According to the assessment of Art. 44 para. 3 Directive 2001/82/EC, the import of (cell-based) veterinary pharmaceuticals into the EU from countries that are not members of the EU is subjected to an official authorization. The competence for issuing such an authorization follows from the legal provisions of the country into which the (cell-based) veterinary pharmaceutical is to be imported as well as from EU provisions. The import license is limited to the respective country and not to be confused with the regular marketing authorization of a veterinary pharmaceutical under pharmaceutical law. As a rule, such an import license is linked to the condition that no authorized pharmaceutical is on the market within the EU for the respective animal species and/or indication.

When importing animal cells – regardless of their pharmaceutical nature – from non-EU Member States into the EU, caution is generally advised. Animal cells can principally not be imported freely into the EU. There are specific import regulations, such as EU (EC) Regulations No. 1069/2009 or No. 142/2011, to be observed. The purpose of these regulations is, among others, to avoid the spreading of animal epidemics within the EU.

## Cross-Border Movement of Cell-Based Veterinary Pharmaceuticals within the European Union

In a Member State of the EU, a (cell-based) veterinary pharmaceutical may only be placed on the market – as stipulated in Art. 5 para. 1 Directive 2001/82/EC – if a marketing authorization has been granted by the competent authorities of that Member State in accordance with this Directive, or if a marketing authorization has been granted in accordance with Regulation (EC) No. 726/2004. Only those veterinary pharmaceuticals placed on the market in accordance with Regulation (EC) No. 726/2004, i.e., those authorized centrally by the EU, may be transferred across borders within the EU without further Member State authorization. If the respective (cell-based) veterinary pharmaceutical, however, is on the market in a Member State under a Member State authorization, it is permitted to be on the market only in that Member State. The transfer of such a veterinary pharmaceutical from the Member State in which its marketing is authorized into Member State in which its marketing is not authorized is then governed by the legal provisions of the country into which the pharmaceutical is to be transferred.

In case of violations of the authorization and marketing authorization requirements, the veterinarian cannot claim to have been unaware of these requirements. Basic knowledge of whether the pharmaceuticals he/she uses conform to authorization requirements is one of the core competences of a practicing veterinarian. If the veterinarian is uncertain about the conformity to authorization requirements, he/she can be expected to inform him/herself of the authorization status of a specific pharmaceutical before using it. Violations of requirements under pharmaceutical law and thus under the codes of professional conduct of veterinarians can cast doubt on the reliability of the veterinarian and thus the legitimacy of his/her medical license where this is provided for in the individual Member State legislation.

## Cell Therapy Products for Food-Producing Animals

To ensure the quality of the food and its safety for human consumption, the use of medicinal products in food-producing animals is subjected to more stringent requirements than the use of medicinal products in non-food-producing animals (cf. Art. 6 and 66 para. 3 Directive 2001/82/EC). This principle thus has to apply to cell-based veterinary pharmaceuticals as well, regardless of whether such cell-based veterinary medicinal products are marketed in the sense of pharmaceutical law or are veterinary pharmaceuticals manufactured individually by veterinarians. The important factor is the use of the pharmaceutical. The only medicinal products permitted to be used in food-producing animals are those that have either undergone a positive residue assessment [cf. Art. 6 Directive 2001/82/EC as well as EU Regulation (EC) No. 37/2010] or out-of-scope-substances within the meaning of Regulation [(EC) 470/2009], which are exempted from the requirement of a residue assessment. Out-of-scope-substances are substances or their residues, which do not pose danger to the health of the consumer on the basis of currently known facts. Although such residue assessments do not yet exist for cell-based veterinary pharmaceuticals, stem cells in general are meanwhile listed as out-of-scope. Therefore, at least stem cells can be used within autologous and allogenic treatments for food-producing animals. It has not yet been finally clarified whether this out-of-scope requirement regarding stem cells also includes differentiated cells (e.g., chondrocytes either biopsied themselves and then transplanted or differentiated from stem cell). In any case, the out-of-scope requirements only exempts from the residue assessment, but not from the requirement for the market authorization.

## Cell Therapy Products for Food-Producing Equidae

The exceptions applying to food-producing Equidae differ from those for other food-producing animals, as stipulated in Art. 10 para. 3 Directive 2001/82/EC. First, unless otherwise specified in the horse passport, Equidae may be treated with medicinal products that may be administered to other food-producing animals as well. Second, food-producing Equidae may additionally be treated with certain medicinal products that are not listed in the appendix of Regulation (EC) No. 37/2010 and consequently do not require a residue assessment. This presupposes, however, that such medicinal products contain substances, which EU Regulation No. 1950/2006 [in current version following modifications by Regulation (EU) Nr. 122/2013], in accordance with Directive 2001/82/EC (Art. 10 para. 3), lists as substances that are essential for the treatment of Equidae. But these so-called whitelists so far do not contain any cell-based pharmaceuticals either, making the use of cell-based pharmaceuticals incompatible with the status of a food-producing animal. However, since stem cells have already out-of-scope status according to Regulation (EC) 470/2009 for food-producing animals in general, stem cells can also be used to treat food-producing Equidae; inasmuch as such stem cell-based medicinal products are in compliance with the further pharmaceutical legislation, e.g., the requirement for market authorization (cf. Cell Therapy Products for Food-Producing Animals).

## Additional Problem: Use of Genetic Engineering Processes for Veterinary Pharmaceuticals That have Not been Authorized Under Pharmaceutical Law

The use of cell-based veterinary pharmaceuticals containing genetically modified organisms (GMOs) in the sense of the European genetic engineering law in an animal may entail additional legal requirements under the genetic engineering law. Regarding cell-based veterinary pharmaceuticals, the requirements of Release Directive 2001/18/EC and of System Directive 2009/41/EC or their acts of transposition in the respective Member States may be of significance. The System Directive regulates the handling of GMOs within genetic engineering facilities such as laboratories. The Release Directive specifies, among others, the legal conditions for field tests and certain types of marketing of GMOs.

The applicability of the Genetic Engineering Act to the field of veterinary therapy further ensues from the fact that the animal treated with the genetically modified cells becomes itself a genetically modified (whole) organism in the sense of the Genetic Engineering Act by the transfer of these cells. This legal assessment follows from Art. 2 No. 2 Sentence 1 Directive 2001/18/EC regarding the definition of “genetically modified organism” and from Art. 2 Sentence 2 No. 2 lit. a); Annex I A, Part 1 Directive 2001/18/EC regarding the definition of genetic modification techniques.

Art. 2 Sentence 2 No. 2 lit. a); Annex I A, Part 1 Directive 2001/18/EC contains a non-exhaustive – “*inter alia*” – list of genetic modification techniques in the sense of the Genetic Engineering Act. This means that under legal aspects the creation of a GMO can also occur, if the specific technique is not explicitly mentioned in the law, but if the assessment made by the law in the specific cases given can also be applied to unmentioned techniques. According to Nos. 1, 2 Annex I A, Part 1 concerning Art. 2 Sentence 2 No. 2 lit. a) Directive 2001/18/EC, techniques by which genetic material produced outside the organism and thus not naturally occurring in this organism is inserted into an organism that has not been genetically modified, are considered genetic modifications, so that the organism into which this non-natural genetic material is inserted is regarded as a GMO. In addition, No. 3 Annex I A, Part 1 concerning Art. 2 Sentence 2 No. 2 lit. a) Directive 2001/18/EC classifies the fusion of a genetically modified cell (=GMO as per Art. 2 No. 2 Sentence 1 Directive 2001/18/EC) with a naturally occurring cell as the formation of a genetically modified (whole) organism. If therefore even one genetically modified cell is transferred to a genetically not modified animal (=organism in the sense of the Genetic Engineering Act), the animal is regarded as a genetically modified (whole) organism after that transfer. It cannot be ruled out in this case that the modified genetic information of the transferred cell may also pass entirely or partially to other cells of the animal after the transfer. Nor is the transfer of genetically modified cells to a genetically not modified organism therefore of a different quality, under the aspect of genetic modification, than the fusion of a genetically modified cell with a genetically not modified cell, a technique regarded as genetic modification according to Nos. 1, 2 Annex I A, Part 1 concerning Art. 2 Sentence 2 No. 2 lit. a) Directive 2001/18/EC.

Art. 5 Directive 2001/18/EC finally regulates the release and marketing of GMOs and of products containing GMOs or components thereof. Since an animal treated with genetically modified cells is itself a GMO in the sense of the Genetic Engineering Act, the release of such an animal from the genetic engineering facility (Art. 4 para. 1, Art. 5 ff. Directive 2001/18/EC) as well as the marketing (=transfer to third parties, as in the case of release from the veterinarian’s office and transfer to the animal’s owner) (Art. 4 para. 1, Art. 12 ff. Directive 2001/18/EC) would require authorization. Besides, the veterinarian who wishes to prepare and/or use genetically modified cells would have to maintain a genetic engineering facility in the sense of the genetic engineering law (cf. Art. 1 Directive 2001/18/EC) that would have to be officially reported or registered, depending on the genetic engineering performed, which could require authorization itself.

The same applies if the veterinarian wants to market merely the genetically modified cells needed for autologous or allogenic treatment models. This is rated as the marketing of products containing or consisting of GMOs, which is subjected to authorization under Art. 4 para. 1, Art. 12 ff. Directive 2001/18/EC. Aside from this authorization requirement pursuant to the Genetic Engineering Act, such actions would probably result in veterinary pharmaceuticals that require marketing authorization because the cells concerned would be marketed in the sense of the pharmaceutical law.

Violations of the requirement to obtain authorization for the operation of a genetic engineering facility and violations relative to a release can be prosecuted under national law where this is provided for in the individual Member State legislation as it is, for example, within the German Genetic Engineering Act and with the German Criminal Code.

## Conclusion and Outlook

Techniques of regenerative medicine, such as cell-based techniques or the use of cell-free preparations made from blood or bone marrow, take the therapeutic efforts of veterinarians to new dimensions and are therefore sought after and put to use (1, 2). In any case, the practice of sending tissue such as bone marrow, fat, or blood as well as (stem) cells to a laboratory to get a usable product in return, or of acquiring finished products from national or even international suppliers is a legal minefield for the veterinarian, in as much as it is unclear for the veterinarian whether the third-party manufacturer holds an official EU law compliant manufacturing license. It is necessary, however, to create legal provisions that will help solve this unsatisfactory condition. Veterinary pharmaceutical law is currently being reformed on the European level. In its position paper of July 15, 2010 (12), the EMA stated that cell-based veterinary pharmaceuticals as the so-called advanced therapy veterinary medicinal products are not yet covered by the EU code for veterinary pharmaceuticals (Directive 2001/82/EC). There is a need, however, for a uniform legal framework throughout the EU for such advanced therapy veterinary pharmaceuticals, similar to the ATMP regulation on advanced therapies, which apply to advanced therapy pharmaceuticals for human use. This idea is also taken up by the “Proposal for a Regulation of the European Parliament and of the Council on veterinary medicinal products, COM(2014)558 final” prepared in September 2014. According to Art. 38 No. 2 lit. d) of the proposed regulation, biological veterinary pharmaceuticals containing or consisting of artificially produced allogenic tissue or cells would then have to be authorized centrally by the EU. Only those veterinary pharmaceuticals containing autologous or allogenic cells or tissue that were not subjected to any industrial process would not have to be centrally authorized by the EU, according to Art. 2 para. 4 lit. b) of the proposed regulation. This raises the further question, though, whether they need to be authorized by the individual Member States before being permitted to be placed on the market. Finally, it is to be welcomed that in 2014 the EMA has established the “*Ad Hoc* Expert Group on Veterinary Novel Therapies” (ADVENT) to provide advice to the Committee for Medicinal Products for Veterinary Use (CVMP) within the EMA (13). Since the concepts of cell-based therapies in veterinary as well as in the human sector are novel therapies compared to classically chemical pharmaceuticals, the ADVENT will also work on cell-based therapies for veterinary use (14).

## Author Contributions

Prof. WB initiated the work on this subject, describing the basic situation of stem cell therapy in horses, and constantly worked on the text. Dr. TF is a Biologist and Lawyer (Dr. iur.) and a specialist in stem cell law and pharmaceutical law. He worked out the legal aspects of veterinary stem cell therapy and, therefore, is the corresponding author.

## Conflict of Interest Statement

The authors declare that the research was conducted in the absence of any commercial or financial relationships that could be construed as a potential conflict of interest.

## Footnotes

^1^So at least even for human organs, so that it can safely be assumed for animal organs as well (6), see also Ref. (7), which states that the organ removal does not (yet) have to follow GMP conditions.

^2^All citations relative to medicinal products for human use. But there can be no legal difference for veterinary medicinal products since the terms “dispensing/placing on the market” are treated identically for medicinal products for veterinary and for human use at least within the German Medicinal Products Act.
